# Maladaptive Decision Making in Adults with a History of Adolescent Alcohol use, in a Preclinical Model, Is Attributable to the Compromised Assignment of Incentive Value during Stimulus-Reward Learning

**DOI:** 10.3389/fnbeh.2017.00134

**Published:** 2017-07-25

**Authors:** Lauren C. Kruse, Abigail G. Schindler, Rapheal G. Williams, Sophia J. Weber, Jeremy J. Clark

**Affiliations:** ^1^Department of Psychiatry and Behavioral Sciences, University of Washington Seattle, WA, United States; ^2^Geriatric Research Education and Clinical Center, VA Puget Sound Health Care System Seattle, WA, United States; ^3^Graduate Program in Neuroscience, University of Washington Seattle, WA, United States

**Keywords:** adolescent, alcohol, decision making, incentive learning, risk taking, impulsivity

## Abstract

According to recent WHO reports, alcohol remains the number one substance used and abused by adolescents, despite public health efforts to curb its use. Adolescence is a critical period of biological maturation where brain development, particularly the mesocorticolimbic dopamine system, undergoes substantial remodeling. These circuits are implicated in complex decision making, incentive learning and reinforcement during substance use and abuse. An appealing theoretical approach has been to suggest that alcohol alters the normal development of these processes to promote deficits in reinforcement learning and decision making, which together make individuals vulnerable to developing substance use disorders in adulthood. Previously we have used a preclinical model of voluntary alcohol intake in rats to show that use in adolescence promotes risky decision making in adulthood that is mirrored by selective perturbations in dopamine network dynamics. Further, we have demonstrated that incentive learning processes in adulthood are also altered by adolescent alcohol use, again mirrored by changes in cue-evoked dopamine signaling. Indeed, we have proposed that these two processes, risk-based decision making and incentive learning, are fundamentally linked through dysfunction of midbrain circuitry where inputs to the dopamine system are disrupted by adolescent alcohol use. Here, we test the behavioral predictions of this model in rats and present the findings in the context of the prevailing literature with reference to the long-term consequences of early-life substance use on the vulnerability to develop substance use disorders. We utilize an impulsive choice task to assess the selectivity of alcohol’s effect on decision-making profiles and conditioned reinforcement to parse out the effect of incentive value attribution, one mechanism of incentive learning. Finally, we use the differential reinforcement of low rates of responding (DRL) task to examine the degree to which behavioral disinhibition may contribute to an overall decision-making profile. The findings presented here support the proposition that early life alcohol use selectively alters risk-based choice behavior through modulation of incentive learning processes, both of which may be inexorably linked through perturbations in mesolimbic circuitry and may serve as fundamental vulnerabilities to the development of substance use disorders.

## Introduction

Adolescence is characterized by risky and impulsive behaviors, often including initial experimentation with drugs of abuse and most commonly, alcohol (Casey and Jones, 2010). During development, the adolescent brain undergoes substantial maturation in cortical and limbic regions involved in impulsive behaviors, decision making and reward processing (Spear, 2000; Chambers et al., 2003; Crews et al., 2007; Bava and Tapert, 2010; Marinelli and McCutcheon, 2014). Given the high vulnerability of these developing regions to the damaging effects of alcohol (Chambers et al., 2003; Pascual et al., 2009; Bava and Tapert, 2010), exposure during this critical time period can produce long-term neurobiological and behavioral changes that increase risk for chronic alcohol problems later in adulthood. In clinical work, alcohol use has been associated with deficits in adaptive decision making strategies, impulsivity and reward valuation (Goudriaan et al., 2007; Johnson et al., 2008; Brevers et al., 2014).

Using a probability discounting task coupled with a preclinical model of voluntary adolescent alcohol use, our laboratory previously demonstrated that rats with a history of adolescent alcohol use opt for more risky decision-making strategies when they are adults, consistent with findings in humans (Brevers et al., 2014; Schindler et al., 2014). This increase in risk preference is specific to adolescent alcohol use as adult animals with identical alcohol exposure do not differ in the probability discounting task (Schindler et al., 2014). Subsequent reports have demonstrated that this behavioral phenotype following adolescent alcohol use is robust (Nasrallah et al., 2011; Clark et al., 2012; Schindler et al., 2014), reproducible (Boutros et al., 2014; McMurray et al., 2014), and translates to the human condition (Brevers et al., 2014). These three core features: robust, reproducible and translational make up the fundamental elements of a successful pre-clinical model of attributes that may correspond to vulnerabilities to the development of substance abuse and alcohol use disorders (Collins and Tabak, 2014).

In addition, we have identified a neural correlate of maladaptive decision making in the mesolimbic dopamine system that is promoted by early life alcohol use. Adult animals with a history of alcohol use in adolescence show increased phasic dopamine release in the nucleus accumbens core in response to risky options and pavlovian incentive cues when compared to controls (Nasrallah et al., 2011; Spoelder et al., 2015). The mesolimbic dopamine system is implicated in reinforcement learning, goal-directed behavior and motivational processes, and may be particularly critical when these behaviors are associated with the craving and seeking of abused substances (Kelley, 2004; Everitt and Robbins, 2005; Salamone and Correa, 2012). Further examination of this mesolimbic circuit revealed a potentiation in the excitatory drive on ventral tegmental area dopamine neurons from the pedunculopontine nucleus (PPT) in animals with a history of adolescent alcohol use (Schindler et al., 2016). This is particularly striking given the important role that this structure is thought to play in both incentive-based reinforcement learning (Pan and Hyland, 2005) and choice behavior under uncertainty (Leblond et al., 2014). Importantly, we were able to demonstrate that pharmacological mitigation of this increased PPT drive was capable of completely rescuing maladaptive risk preference in animals with a history of adolescent alcohol use (Schindler et al., 2016). We have therefore hypothesized that maladaptive decision making under risk as a result of adolescent alcohol use perturbing the mesolimbic dopamine system may result from a selective deficit in incentive learning (Clark et al., 2012; Spoelder et al., 2015).

However, both risk-seeking and risk-taking behaviors have been previously attributed to impulsivity, which is another potential underlying mechanism influencing altered decision making. The concept of impulsivity incorporates various behaviors, but is commonly separated into two important features: impulsive choice and impulsive action, both of which are linked to addiction (Evenden, 1999; Olmstead, 2006; Winstanley et al., 2010). Indeed, preclinical and clinical studies have long identified impulsivity and risky decision making as behaviors contributing to the development and perpetuation of substance use disorders, including alcohol (Littlefield and Sher, 2010; Brevers et al., 2014; Jupp and Dalley, 2014a,b). Delay discounting is commonly used as a measure of impulsive decision making and performance on this task has historically been linked with performance on probability discounting tasks (Richards et al., 1999). In addition to the temporal delay of rewards influencing decision making, the amount of effort required to receive a reward can also influence choice. Several studies have connected dopamine signaling with mediating choice in the effort discounting task, but the role of alcohol, and particularly adolescent alcohol use, on effort discounting remains unclear (Floresco et al., 2008; Day et al., 2010; Ghods-Sharifi and Floresco, 2010). Interestingly, recent reports propose that performance on delay discounting and probability discounting require different decision-making processes with separate underlying neurobiological mechanisms (van Gaalen et al., 2006; Olson et al., 2007; St Onge and Floresco, 2009; Green and Myerson, 2013). In the present studies, we examine the effect of voluntary adolescent alcohol intake on impulsivity and incentive attribution to differentiate the potential role impulsivity may play in risk preference from the role incentive learning may play.

We examined differences in incentive attribution as a result of adolescent alcohol intake using Pavlovian conditioning and conditioned reinforcement, the gold standard for examining acquired motivational properties through learning. We also examined the two primary features of impulsivity, impulsive choice and impulsive action, using delay-discounting and the differential reinforcement of low rates of responding (DRL) tasks, respectively. The purpose of these studies is to further test our hypothesis that incentive based learning and assignment of motivational value through reinforcement learning, not impulsivity, is the underlying psychological mechanism of maladaptive decision making after early life alcohol use. Our results show that adults exposed to alcohol during adolescence exhibit enhanced attribution of value to reward-predictive cues compared to controls. Alternatively, we find no significant difference between alcohol-exposed and control animals on either measure of impulsivity (impulsive choice and action). Therefore, we provide further compelling evidence that adolescent alcohol use promotes disadvantageous decision making by altering a learning-based incentive attribution process and not through a general mechanism of impulsivity.

## Materials and Methods

### Animals and Housing

Male Sprague-Dawley rats (Charles River, Hollister, CA, USA) were weaned at postnatal day (PND) 21 and aged PND 27 at the start of experiments. All animals were housed individually in polycarbonate tubs on a 12-h light/dark cycle (lights on at 06:00). Animals were housed individually for accurate assessment of alcohol gel consumption. Water and Teklad (Harlan, Kent, WA, USA) rodent chow was available* ad libitum* except as noted. Rats were weighed and handled daily throughout the course of the experiment. All work in this manuscript was conducted under the guidance and permission of the Institutional Animal Care and Use Committee at the University of Washington and pursuant to federal regulations regarding work with animals.

### Alcohol Preparation, Administration and Withdrawal

Alcohol was presented to adolescent (PND 30–49) rats in a gel matrix consisting of distilled water, Knox gelatin, polycose (10%) and 190-proof ethanol (10%). Control gels had ethanol replaced with distilled water. Preparation was as previously described (Rowland et al., 2005; Nasrallah et al., 2011; Schindler et al., 2014). The gels were made available 24 h/day, unless otherwise noted, in addition to *ad libitum* water and chow. Alcohol gel intake levels were monitored daily and expressed in g/kg of body weight using individual gel consumption and body weights measured daily. Rats failing to consume gel during the control gel pre-exposure, exhibiting three consecutive days of no consumption, or burying of the gel in bedding once the alcohol gel exposure began were excluded from the study. Experiments began with 3 days of pre-exposure to control gel. Subsequently, adolescent rats were each split into alcohol gel and control gel groups matched by weight and baseline intake. Twenty days of 24 h/day gel exposure followed. Upon completion of the 20-day exposure to ethanol rats were monitored daily for withdrawal symptoms (e.g., seizures, weight loss and anxious behavior) for the following 20 days. No overt signs of withdrawal were observed.

### Instrumental Training

All behavioral experiments were conducted in standard Med Associates chambers (St. Albans, VT, USA). For all tasks, prior to the beginning of training, animals were food restricted to maintain them at ~90 ± 2% free-feeding weight. Free-feeding weight was based on pre-restriction weights. The rewards for instrumental responses and during Pavlovian conditioning were 45-mg sucrose pellets (Bio Serve, Prospect, CT, USA). Rats underwent magazine training before all tasks where 10 sucrose pellets were delivered over 15 min. For delay discounting and DRL, rats were trained on a fixed ratio 1 schedule to a criterion of ≥24 responses in a 30-min session. For delay discounting, once criterion was met, rats were auto-shaped over the course of 5 days (day 1 of auto-shaping required rats to perform a nosepoke into the food tray for trial initiation; day 2 increased the inter-trial interval (ITI) from 0 s to 15 s; day 3 reduced the time to perform trial-initiating nosepoke to 10 s; day 4 increased the ITI from 15 s to 30 s; day 5 increased the ITI from 30 s to 45 s).

### Pavlovian Conditioned Approach and Conditioned Reinforcement

Following magazine training, rats underwent 7 days of pavlovian training with 25 trials per day. For each training session, a trial consisted of the extension of a lever (left or right, counterbalanced between animals) into the chamber along with illumination of a cue light above the lever (conditioned stimulus, CS) for 8 s, followed immediately by the delivery of two sucrose pellets (unconditioned stimulus, US) into the food tray and illumination of the tray light. The CS-US presentations occurred on a 60 s variable ITI schedule for each session. Lever presses and cup entries during lever presentation were recorded as measures of pavlovian conditioned approach. Total number of lever presses and food tray head entries during lever presentation were recorded but had no programmable response.

After 7 days of pavlovian training, all animals underwent a test for conditioned reinforcement. The chambers were rearranged such that the left and right retractable levers flanking the food tray were replaced with nosepoke ports and the food tray was replaced with a single retractable lever. The conditioned reinforcement session lasted a total of 40 min and the houselight was on for the full duration. During the session, nosepokes into the designated “active” port (located on the opposite side of wall as the lever-CS) resulted in insertion of the illuminated lever for 2 s. Nosepokes into the “inactive” port had no programmable consequences. Total number of lever presses, nosepokes into the active port, and nosepokes into the inactive port were recorded.

### Delay Discounting

Following instrumental training as outlined above, animals were tested on a concurrent instrumental task involving the presentation of two levers. Each daily session consisted of 24 forced trials followed by 24 free-choice trials, with a total of seven testing sessions. At the start of each session, the chamber was in the ITI state, completely dark with no light cues. All trials began with illumination of the house light and a light in the food tray cueing the animal to make a nosepoke into the food tray within 10 s. Failure to nosepoke resulted in trial termination, and the chamber returned to the ITI state. During training, animals were exposed to forced trials wherein a successful nosepoke led to the extension of a single lever, presented pseudo-randomly. These trials served to expose the animal to each option and its associated expected value. During each session, forced choice trials were followed by free-choice trials with the same contingency for each lever. Free-choice trials follow the guidelines described above, but each successful nosepoke resulted in the extension of both levers, and the animal was free to choose between the two levers within 10 s. During both forced- and free-choice trials, one lever was associated with the delivery two sucrose pellets after 1 s and the other lever associated with the delivery of four sucrose pellets after an increasing delay of time (either 1, 2, 4, 8, 16, 32 and 64 s). Increasing delay times were tested in separate testing sessions (days) with a 1 s delay on the first day of testing, a 2 s delay on the second day of testing, and so on for all animals. This method of testing the different delay times on separate days was done in order to be kept consistent with our previous studies using the probability discounting task where the different probabilistic deliveries of reward were also tested on separate, subsequent days. Each session assessed the animal’s preference between the two levers. Choice of the high reward lever was recorded during free-choice trials.

### DRL

Following instrumental training as described above, animals were tested on the DRL task as a measure of impulsive action. Each 45 min session began with illumination of the house light and lever presentation. Each animal was assigned an active lever (left or right) counterbalanced between rats, which remained extended throughout the duration of the sessions. For the first 5 days, rats were tested using a 5 s DRL interval, in which a lever press resulted in pellet delivery only if at least 5 s elapsed since the previous press. Each lever press that occurred before the required 5 s wait period resulted in a resetting of the 5 s criteria, during which animals would have to wait another 5 s before pressing the lever to receive a pellet. Following five consecutive days of the 5 s DRL, the schedule was then switched to a 10 s DRL interval for 5 days, then to a 20 s DRL interval for 5 days, and finally to a 30 s DRL interval for 5 days. The total number of lever presses and the total number of reinforcers received were recorded. Percent of effective lever presses was also determined by dividing the total number of pellets received by total number of lever presses.

### Statistical Analyses

All statistical analyses were conducted using GraphPad Prism 6. Behavioral data for the pavlovian training sessions were binned into five-trial epochs. A response bias score, which is a measure of the relative allocation of behavioral responses between the lever and food tray during pavlovian training, was calculated by subtracting the number of food tray entries from the number of lever presses divided by the sum of both responses: (lever press − food tray entries/(lever presses + food tray entries), resulting in a number ranging from −1 (goal-tracking response) to +1 (sign-tracking response; Meyer et al., 2012). Animals with a response bias >+0.70 were defined as animals with a strong sign-tracking bias and animals with a response bias <−0.70 were defined as having a strong goal-tracking bias, consistent with previous work (Flagel et al., 2011; Meyer et al., 2012; Spoelder et al., 2015). Conditioned responses (lever presses and food tray entries) from all phases of training were analyzed using repeated measures two-way analysis of variance (ANOVA) with treatment (alcohol and control) and trial bin treated as independent variables. Performance on the conditioned reinforcement test was analyzed using a two-way ANOVA with treatment (alcohol vs. control) and port (active vs. inactive) as the independent variables and the number of nosepokes as the dependent variable. A *t*-test was used to analyze total number of lever presses during the conditioned reinforcement test. Performance on the delay-discounting and DRL tasks were analyzed using repeated measures two-way ANOVA with treatment (alcohol vs. control) and delay (1 s, 2 s, 4 s, 8 s, 16 s, 32 s, and 64 s) or DRL interval (5, 10, 20, 30 s) as the independent variables, and preference for the high reward lever or lever presses, reinforcers and effective lever presses, respectively, as the dependent variables. Bonferroni *post hoc* analyses were used and were corrected for multiple comparisons. All data are presented as mean ± SEM and threshold for statistical significance was set at *p* < 0.05.

## Results

### Adolescent Alcohol Intake

For the conditioned reinforcement, delay-discounting and DRL animals, average alcohol intake across the 20-day exposure period was 11.3 ± 1.6 g/kg, 12.0 ± 2.3 g/kg, and 15.4 ± 1.5 g/kg, respectively, and intake did not differ across behavior groups (*F*_(2,30) group_ = 2.1, NS). Across all three behavioral experiments, a total of 10% of rats did not consume the alcohol gelatin during the 20 exposure days and thus were not run in the behavioral studies. These intake levels are comparable to our previous studies further supporting this method of alcohol intake as a consistent model of moderate/recreational alcohol use (Nasrallah et al., 2011; Schindler et al., 2014; Spoelder et al., 2015). Blood alcohol levels were not obtained but have been assessed previously (Schindler et al., 2014).

### The Effect of Adolescent Alcohol Intake on Acquisition of Pavlovian Conditioned Approach Behavior in Adulthood

**Figure 1:**
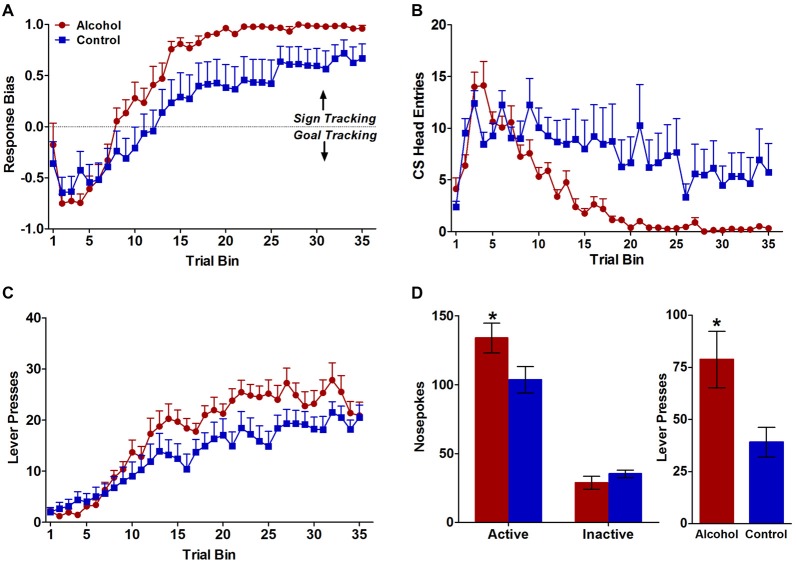
Behavioral responses during pavlovian conditioned approach and test for conditioned reinforcement. **(A)** Alcohol-exposed (*n* = 16) animals reduced their conditioned responses to the food tray, whereas control (*n* = 15) animals continued to approach the food tray throughout the course of learning. **(B)** Both alcohol-exposed and control animals increased their conditioned responses to the reward-predicting lever over the course of learning but alcohol-exposed animals demonstrated a stronger conditioned response to the lever throughout the course of learning. **(C)** Both alcohol-exposed and control animals developed a response-bias toward sign tracking over the course of learning, but alcohol-exposed animals developed a stronger sign-tracker response bias. **(D)** In the test for conditioned reinforcement alcohol-exposed animals designated as sign trackers (*n* = 12) made significantly more active nosepokes compared to controls (*n* = 9). Alcohol-exposed and control animals did not differ in inactive nosepokes. All data are presented as mean ± SEM. *P < 0.05.

Consistent with previously published data (Spoelder et al., 2015), pavlovian conditioned responses to either the reward-predicting lever (sign tracking) or the food tray (goal tracking) during CS presentation developed differently for alcohol-exposed and control animals over the 7 days of pavlovian training. Although both groups developed a response bias towards sign-tracking over the course of training (*F*_(34,986) trial bin_ = 57.2 *p* < 0.0001; Figure 1A), the alcohol-exposed animals showed a stronger sign-tracking bias relative to controls later in the conditioning trials (*F*_(34,986) trial bin × treatment_ = 2.6, *p* < 0.001; *F*_(1,986) treatment_ = 4.3, *p* < 0.05; Figure 1A). Further, both alcohol-exposed and control animals demonstrated reduced conditioned responding to the food tray over trials (*F*_(34,986) trial bin_ = 14.0, *p* < 0.0001; Figure 1B), but alcohol-exposed animals largely stopped responding to the food tray by trial bin 20 (*F*_(34,986) trial bin × treatment_ = 3.9, *p* < 0.0001; *F*_(1,986) treatment_ = 4.6, *p* < 0.05 Figure 1B). Both alcohol-exposed animals and controls showed increased responses towards the lever over trials (*F*_(34,986) trial bin_ = 40.6, *p* < 0.0001; Figure 1C), but a greater number of contacts upon cue presentation in alcohol-exposed animals compared to controls developed over trial bins (*F*_(34,986) trial bin × treatment_ = 2.0, *p* < 0.001; Figure 1C). Response-bias scores were also calculated as described above in the methods section. Animals with scores greater than +0.70 were designated as sign trackers and animals with scores less than −0.70 were designated as goal trackers. Based on these criteria, 13 out of 16 alcohol-exposed animals were sign trackers and 10 out of 15 control animals were sign trackers. Of the remaining eight animals, three alcohol-exposed and five controls had response bias scores that fell between −0.70 and +0.70 and therefore did not show a strong enough response to be classified as sign or goal trackers.

### The Effect of Adolescent Alcohol Intake on Conditioned Reinforcement in Adulthood

All animals underwent the test of conditioned reinforcement following pavlovian training. However, given that this task measures the effectiveness of the lever-CS as a reinforcer (defining a strong sign-tracker bias), we were primarily interested in the behavior of sign-tracking animals. Therefore, only the 23 animals designated as sign trackers based on the above mentioned criterion (>+0.70 response bias score) were kept in the analyses. One sign tracker from each treatment group was dropped because the incorrect active port was assigned for the conditioned reinforcement test yielding *n* = 12 alcohol sign-trackers and *n* = 9 control sign-trackers for the final analyses. During the test of conditioned reinforcement, alcohol rats displayed more incentive attribution to the lever-CS compared to control rats. Specifically, both treatment groups made more active than inactive nosepokes (*F*_(1,38) port_ = 116.4, *p* < 0.0001; Figure 1D), but sign trackers with a history of adolescent alcohol intake demonstrated significantly more nosepokes in the active port compared to controls (*F*_(1,38) treatment × port_ = 5.2, *p* < 0.05; Figure 1D), with no difference between alcohol and control animals in the inactive port confirmed by follow-up tests (*p* < 0.05). Further, during the 2-s lever extension period following a poke in the active port, alcohol animals also had significantly more lever presses than control animals (*t*_(1,19)_ = 2.4, *p* < 0.05; Figure 3). As expected, the alcohol (*n* = 3) and control (*n* = 5) rats that were not classified as sign trackers (response bias scores between −0.70 and +0.70) did not differ in total number of active or inactive nosepokes (*F*_(1,4) treatment_ = 0.2, NS; *F*_(1,4) treatment × port_ = 0.02, NS; Figure 1). Taken together with the results from Pavlovian conditioned approach, our data indicate that the lever-CS acquired both the attractiveness and wanting properties of an incentive stimulus to a greater extent in the alcohol-exposed animals compared to controls.

**Figure 2:**
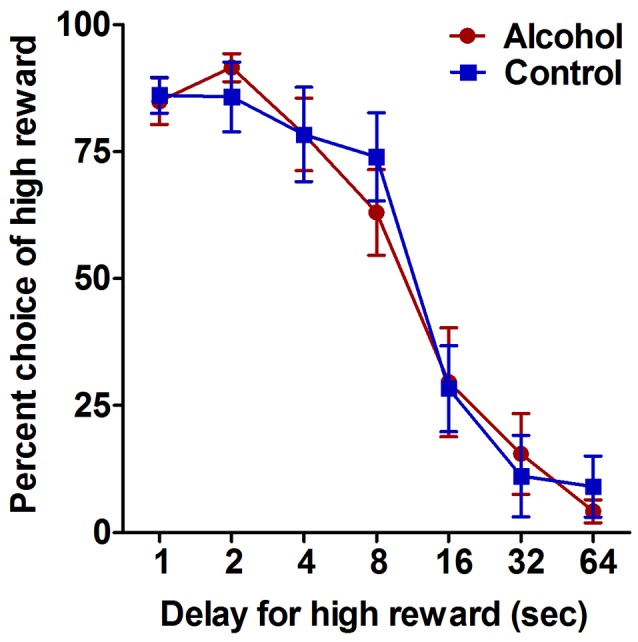
Impulsive choice as measured by performance on the delay-discounting task. Alcohol-exposed (*n* = 7) and control (*n* = 6) animals both demonstrated a decrease in choice of the large reward option with increasing delay. Alcohol-exposed and control animals did not differ in choice behavior over delay conditions. All data are presented as mean ± SEM for the percent of trials on which the larger delayed reward was chosen for 1, 2, 4, 8, 16, 32 and 64 s delay intervals.

**Figure 3:**
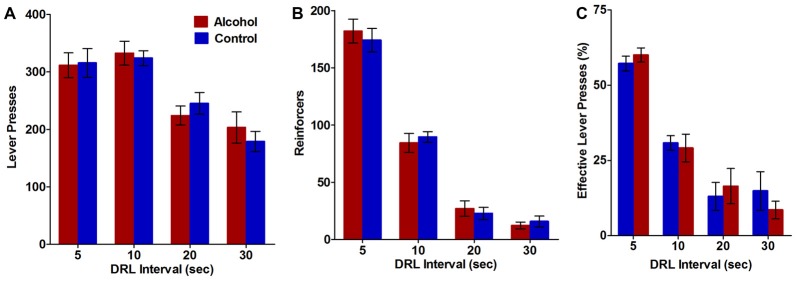
Impulsive action as measured by performance on the differential reinforcement of low rates of responding (DRL) task. Alcohol-exposed (*n* = 9) animals did not differ from controls (*n* = 11) on **(A)** the total number of lever presses performed, **(B)** the total number of reinforcers received, or **(C)** the percentage of lever presses that resulted in reward delivery. All data are presented as mean ± SEM averaged across all 5-day blocks for the 5, 10, 20 and 30 s DRL intervals.

### The Effect of Adolescent Alcohol Intake on Impulsive Choice in Adulthood

The delay-discounting task was used to assess the effect of adolescent alcohol intake on impulsive choice in adulthood. Analysis of choice behavior on the delay-discounting task generated standard discounting curves for choice of the larger but delayed reward option over all conditions, with increasing delay of the large reward resulting in decreased choice of the large reward option for both alcohol-exposed and control animals (*F*_(6,66) delay_ = 62.1, *p* < 0.0001; Figure 2). Alcohol-exposed and control animals did not differ in choice behavior over any of the delay conditions (*F*_(1,66) treatment_ = 0.02, NS; *F*_(6,66) delay × treatment_ = 0.4, NS; Figure 2). These data indicate that moderate adolescent alcohol intake does not appear to alter impulsive choice in adulthood compared to animals without a history of adolescent alcohol.

### The Effect of Adolescent Alcohol Intake on Impulsive Action in Adulthood

The DRL task was used to assess the effect of adolescent alcohol intake on impulsive action in adulthood, as measured by the ability of the animals to withhold responding for reinforcers during 5, 10, 20, and 30 s DRL intervals. Alcohol-exposed animals did not differ from controls on total number of lever presses (*F*_(3,54) DRL × treatment_ = 0.6, NS; *F*_(1,54) treatment_ = 0.01, NS; Figure 3A) or total number of reinforces received (*F*_(3,54) DRL × treatment_ = 0.4, NS; *F*_(1,54) treatment_ = 0.02, NS; Figure 3B) at any of the DRL intervals. Effective lever presses were also analyzed as a measure of the percent of lever presses that resulted in delivery of the reward. Alcohol treatment during adolescence did not alter the percent of effective lever presses (*F*_(3,54) DRL × treatment_ = 0.7, NS; *F*_(1,54) treatment_ = 0.01, NS; Figure 3C) at any of the DRL intervals. Total lever presses (*F*_(3,54) treatment_ = 24.9, *p* < 0.0001; Figure 3A), reinforcers (*F*_(3,54) treatment_ = 246.8, *p* < 0.0001; Figure 3B), and effective lever presses (*F*_(3,54) treatment_ = 63.9, *p* < 0.0001; Figure 3C) decreased with increasing DRL intervals, in both groups, suggesting animals in both groups were less able to withhold responding as the time to wait before responding increased. Thus, using our model of voluntary alcohol intake, adolescent alcohol use does not appear to differentially affect the ability of animals to withhold responding for a reward compared to controls.

## Discussion

Alcohol is the most commonly abused substance among adolescents and use during this sensitive developmental period is one of the best predictors of risk for development of alcohol use disorder (Grant et al., 2001). Maladaptive decision making strategies and altered reinforcement learning processes are two long-term consequences of adolescent alcohol use that may be contributing factors for alcohol problems later in life. Using a preclinical model of moderate voluntary adolescent alcohol intake, our laboratory has shown that alcohol use during this sensitive developmental period promotes risky decision making and alterations in incentive learning processes in adulthood, both of which are mirrored by perturbations in cue-evoked dopamine signaling (Nasrallah et al., 2011; Schindler et al., 2014; Spoelder et al., 2015). We have proposed that these two processes, risk-based decision making and incentive learning, are fundamentally linked through dysfunction of midbrain circuitry where inputs to the dopamine system are disrupted by adolescent alcohol use; with the latter potentially influencing performance on the former. The present work supports the behavioral predictions of this model. We show that alcohol-exposed animals attribute greater incentive value to a reward-predicting cue, demonstrated by increased approach to the cue and a greater willingness to work to receive the cue even in the absence of reward. Further, we show that animals exposed to alcohol during adolescence did not differ from controls in impulsive choice, impulsive action, or effortful choice. The findings presented here, in combination with previous work from our laboratory, support the proposition that early life alcohol use selectively modulates incentive learning processes and alters risk-based choice behavior (Clark et al., 2012; Schindler et al., 2014; Spoelder et al., 2015), but not other aspects of decision making including impulsive choice and behavioral inhibition.

Cues in an environment that repeatedly predict reward can attain value through a Pavlovian process referred to as stimulus-reward learning. In the realm of addiction, cues associated with drugs often attain enhanced incentive value and play a critical role in promoting drug craving, seeking and relapse following periods of abstinence (O’Brien et al., 1998; Shaham et al., 2003; Milton and Everitt, 2012). In preclinical research, animals classified as sign trackers show enhanced attribution of value to reward predicting cues, are more prone to display behaviors associated with drug-abuse, and are thought to model addiction-vulnerable populations in humans (Flagel et al., 2009, 2010; Spoelder et al., 2015). The value attributed to incentive stimuli can be defined by attractiveness, measured by approach to the stimulus, and wanting, measured by the animal’s willingness to work to obtain the stimulus (Berridge et al., 2009). Consistent with our previous work (Spoelder et al., 2015), we confirm that voluntary adolescent alcohol exposure results in adult animals finding the reward-predicting cue more attractive, compared to control animals, as evidenced by a greater sign-tracking bias and increased approach to the lever-CS. Greater sign-tracking behavior in adulthood following a history of adolescent alcohol has also been shown with non-voluntary models of alcohol administration (McClory and Spear, 2014). Thus, exposure to alcohol during adolescence appears to consistently produce a phenotype associated with addiction-vulnerability. Further, we have previously shown that this greater attribution of value to reward-predictive cues may be dopamine dependent (Spoelder et al., 2015). We show that sign trackers with a history of adolescent alcohol use demonstrated a greater wanting of the cue by working harder than control sign trackers to obtain the lever-CS even in the absence of reward delivery. Alcohol-exposed animals also made significantly more lever presses following an active nosepoke during the 40 min conditioned reinforcement test, further demonstrating the greater attraction of the lever-CS for alcohol-exposed animals compared to controls. These data support evidence in both clinical and preclinical literature that alcohol use during adolescence, whether moderate or heavy, leads to alterations in incentive learning processes producing behaviors often apparent in addiction-prone individuals.

These findings further lend support to our hypothesis that maladaptive decision making under risk, a consequence of adolescent alcohol use, is a result of altered incentive learning. However, other underlying constructs such as altered valuation of primary rewards, deficits in reinforcement learning where corrupted value is assigned to specific cues and actions, and/or increased impulsivity, could also influence the risk-seeking phenotype. Thus, in a series of experiments begun in Clark et al., 2012 and completed in the current work we have attempted to parse the specific contribution of these constructs to maladaptive decision making in adults with a history of adolescent alcohol use. First, we asked if increased risk preference was due to altered reward valuation where the shape of an individual’s utility curve is thought to determine choice under uncertainty (Clark et al., 2012). Indeed, in economic theory, risk attitude is attributed to this relationship between the objective value of a reward and its desirability (subjective value; Glimcher and Rustichini, 2004). Reward valuation was assessed by conducting progressive ratio experiments at different reward levels to estimate each subject’s utility curve, and no differences between alcohol and control groups were found (Clark et al., 2012). We hypothesized, therefore, that increased risk preference can be attributed to a selective deficit in reward learning as probability discounting is a special case where subjects are asked to constantly alter the value they assign to a probabilistic option trial by trial through reinforcement learning. We showed this in a simple computational model and supported this hypothesis in further studies where we demonstrated that alcohol animals, consistent with human alcoholics, are more prone to risky choices in the gain domain and after wins rather than losses (Clark et al., 2012; Brevers et al., 2014; Schindler et al., 2016). Finally, in the current work, we explore impulsivity as a potential contributor to the risk-seeking phenotype.

Deficits in impulse control are a key feature of alcohol use disorders, with higher levels of impulsivity predisposing to the development of alcohol problems later in life and increased likelihood of relapse following abstinence (Mitchell et al., 2005; Jupp and Dalley, 2014b; Stevens et al., 2014). Impulsive choice reflects a more cognitive aspect of impulsivity thought of as impulsive decision making, whereas impulsive action reflects an inability to inhibit a motor response thought of as behavioral inhibition (Evenden, 1999; Olmstead, 2006; Winstanley et al., 2010). Using delay discounting, we show that voluntary alcohol intake during adolescence had no effect on impulsive choice in adulthood, evidenced by similar discounting curves between alcohol-exposed and control animals across delay sessions. These results are consistent with another study that used a non-voluntary model of alcohol administration and found no effect of chronic intermittent ethanol exposure during adolescence on impulsive choice when tested later in adulthood (Mejia-Toiber et al., 2014). Adolescent alcohol exposure also did not alter the ability to withhold responding for a reward in the DRL task, indicating that moderate adolescent alcohol intake does not alter impulsive action in adulthood. Finally, the amount of work required to obtain a reward could also influence decision making under risk. Less is known about the relationship between effortful decision making and alcohol use disorders, but our data show that moderate alcohol use during adolescence does not appear to influence effortful decision making in adulthood (Supplementary Figure S1).

Previous studies have explored the relationship between probability discounting, delay discounting, incentive learning, sign-tracking behavior and addiction-prone phenotypes with varying results on how these behaviors cluster (Flagel et al., 2010; Lovic et al., 2011). For instance, Lovic et al. (2011) found that sign trackers displayed greater impulsive action, but not choice, compared to goal trackers. (Flagel et al. (2010); Flagel et al. (2011)) found that rats selectively bred for response to novelty, a phenotype associated with addiction, are sign trackers that also show greater impulsive action but not choice, and greater incentive value attribution but no difference in risky decision making. Our work demonstrates that alterations in decision making under risk and incentive value attribution cluster together as a result of alcohol use during adolescence. Given that we have shown the pharmacological attenuation of risky behavior (Schindler et al., 2016), future studies examining if this same drug attenuates the enhanced attribution of value to reward-predicting cues would confirm an overlapping mechanism between these behaviors that appear to cluster. Together with our previous work, we can begin to outline an emerging profile of the selective effects of moderate adolescent alcohol use on decision making, with apparent perturbations in risk-based, but not delay- or effort-based, decision making.

In addition to delay discounting, the probability-discounting task has also been used as a measure of impulsive choice (Flagel et al., 2010), with some researchers attributing performance on tasks involving decision making under risk to features of impulsivity (Richards et al., 1999). However, emerging clinical and preclinical data indicate that performance on probability-discounting and delay-discounting tasks are dissociable neurobiologically (van Gaalen et al., 2006; St Onge and Floresco, 2009), genetically (Flagel et al., 2010) and by age (Olson et al., 2007). Our data are consistent with and add to this emerging literature by showing that moderate adolescent alcohol use alters probability but not delay discounting in adulthood. It is interesting to speculate how these results may support a differential sensitivity of the underlying neurobiological mechanisms of probability and delay discounting, to alcohol during development. The pathway that mediates decision making under risk appears to be sensitive to moderate levels of alcohol during the adolescent time period, whereas the pathway that mediates either feature of impulsivity is not (Schindler et al., 2014, 2016). We should consider that impulsivity later in life could be altered by adolescent exposure, but heavy or excessive alcohol use may be required to see an effect. Nevertheless, the system that mediates risky behavior appears to be highly sensitive to even moderate levels of alcohol during development identifying it as an important target. Future studies examining the role of impulsivity and risk in substance use disorders should therefore treat delay and probability discounting as separable tasks that can provide unique information about the relationship between adolescent drug use and later decision making deficits.

Taken together with our previous work, the findings presented here support our hypothesis that the compromised encoding of Pavlovian cues following alcohol exposure in adolescence may underlie suboptimal decision making strategies in adulthood. Our data additionally support delay- and probability-discounting as dissociable decision-making paradigms, and confirm that impulsivity is not likely an explanation for maladaptive decision making under risk in adulthood seen following adolescent alcohol use. One limitation of the present set of experiments is that only the effect of adolescent alcohol intake, but not adult alcohol intake, on incentive value attribution and impulsivity in adulthood was assessed. Thus, we cannot state that the results are specific to adolescent intake. However, we have previously demonstrated that moderate alcohol intake during adulthood does not alter risky decision making (Schindler et al., 2014). Taken together with our previous work (Nasrallah et al., 2011; Spoelder et al., 2015; Schindler et al., 2016), we believe these effects are a result of alcohol on the developing mesolimbic dopamine system and would not expect those changes to occur with adult alcohol exposure. Aberrant changes in risk-based decision making and incentive learning following adolescent alcohol use are associated with perturbations in mesolimbic dopamine neurotransmission enduring into adulthood and the combination of such neurobiological and behavioral deficits may serve as critical vulnerabilities to the development of substance use disorders later in life.

## Author Contributions

LCK performed most of the studies, analyzed the data. LCK and JJC wrote the manuscript. JJC, AGS and LCK conceived the studies and edited the manuscript. RGW performed the delay discounting study. SJW performed analyses for the delay data.

## Conflict of Interest Statement

The authors declare that the research was conducted in the absence of any commercial or financial relationships that could be construed as a potential conflict of interest.

## References

[B1] Bava S. Tapert S. F. (2010). Adolescent brain development and the risk for alcohol and other drug problems. Neuropsychol. Rev. 20, 398–413. 10.1007/s11065-010-9146-6 20953990 PMC2988999

[B2] Berridge K. C. Robinson T. E. Aldridge J. W. (2009). Dissecting components of reward: ‘liking’, ‘wanting’ and learning. Curr. Opin. Pharmacol. 9, 65–73. 10.1016/j.coph.2008.12.014 19162544 PMC2756052

[B3] Boutros N. Semenova S. Liu W. Crews F. T. Markou A. (2014). Adolescent intermittent ethanol exposure is associated with increased risky choice and decreased dopaminergic and cholinergic neuron markers in adult rats. Int. J. Neuropsychopharmacol. 18:pyu003. 10.1093/ijnp/pyu003 25612895 PMC4368879

[B4] Brevers D. Bechara A. Cleeremans A. Kornreich C. Verbanck P. Noël X. (2014). Impaired decision-making under risk in individuals with alcohol dependence. Alcohol. Clin. Exp. Res. 38, 1924–1931. 10.1111/acer.12447 24948198 PMC4115290

[B5] Casey B. J. Jones R. M. (2010). Neurobiology of the adolescent brain and behavior: implications for substance use disorders. J. Am. Acad. Child Adolesc. Psychiatry 49, 1189–1201; quiz 1285. 10.1016/j.jaac.2010.08.017 21093769 PMC3099425

[B6] Chambers R. A. Taylor J. R. Potenza M. N. (2003). Developmental neurocircuitry of motivation in adolescence: a critical period of addiction vulnerability. Am. J. Psychiatry 160, 1041–1052. 10.1176/appi.ajp.160.6.1041 12777258 PMC2919168

[B7] Clark J. J. Nasrallah N. A. Hart A. S. Collins A. L. Bernstein I. L. Phillips P. E. (2012). Altered risk-based decision making following adolescent alcohol use results from an imbalance in reinforcement learning in rats. PLoS One 7:e37357. 10.1371/journal.pone.0037357 22615989 PMC3353889

[B8] Collins F. S. Tabak L. A. (2014). Policy: NIH plans to enhance reproducibility. Nature 505, 612–613. 10.1038/505612a 24482835 PMC4058759

[B9] Crews F. He J. Hodge C. (2007). Adolescent cortical development: a critical period of vulnerability for addiction. Pharmacol. Biochem. Behav. 86, 189–199. 10.1016/j.pbb.2006.12.001 17222895 PMC11646682

[B10] Day J. J. Jones J. L. Wightman R. M. Carelli R. M. (2010). Phasic nucleus accumbens dopamine release encodes effort- and delay-related costs. Biol. Psychiatry 68, 306–309. 10.1016/j.biopsych.2010.03.026 20452572 PMC2907444

[B11] Evenden J. L. (1999). Varieties of impulsivity. Psychopharmacology 146, 348–361. 10.1007/pl00005481 10550486

[B12] Everitt B. J. Robbins T. W. (2005). Neural systems of reinforcement for drug addiction: from actions to habits to compulsion. Nat. Neurosci. 8, 1481–1489. 10.1038/nn1579 16251991

[B13] Flagel S. B. Akil H. Robinson T. E. (2009). Individual differences in the attribution of incentive salience to reward-related cues: implications for addiction. Neuropharmacology 56, 139–148. 10.1016/j.neuropharm.2008.06.027 18619474 PMC2635343

[B14] Flagel S. B. Clark J. J. Robinson T. E. Mayo L. Czuj A. Willuhn I.. (2011). A selective role for dopamine in stimulus-reward learning. Nature 469, 53–57. 10.1038/nature09588 21150898 PMC3058375

[B15] Flagel S. B. Robinson T. E. Clark J. J. Clinton S. M. Watson S. J. Seeman P.. (2010). An animal model of genetic vulnerability to behavioral disinhibition and responsiveness to reward-related cues: implications for addiction. Neuropsychopharmacology 35, 388–400. 10.1038/npp.2009.142 19794408 PMC2794950

[B16] Floresco S. B. Tse M. T. Ghods-Sharifi S. (2008). Dopaminergic and glutamatergic regulation of effort- and delay-based decision making. Neuropsychopharmacology 33, 1966–1979. 10.1038/sj.npp.1301565 17805307

[B17] Ghods-Sharifi S. Floresco S. B. (2010). Differential effects on effort discounting induced by inactivations of the nucleus accumbens core or shell. Behav. Neurosci. 124, 179–191. 10.1037/a0018932 20364878

[B18] Glimcher P. W. Rustichini A. (2004). Neuroeconomics: the consilience of brain and decision. Science 306, 447–452. 10.1126/science.1102566 15486291

[B19] Goudriaan A. E. Grekin E. R. Sher K. J. (2007). Decision making and binge drinking: a longitudinal study. Alcohol. Clin. Exp. Res. 31, 928–938. 10.1111/j.1530-0277.2007.00378.x 17403069 PMC2667377

[B20] Grant B. F. Stinson F. S. Harford T. C. (2001). Age at onset of alcohol use and DSM-IV alcohol abuse and dependence: a 12-year follow-up. J. Subst. Abuse 13, 493–504. 10.1016/s0899-3289(01)00096-7 11775078

[B21] Green L. Myerson J. (2013). How many impulsivities? A discounting perspective. J. Exp. Anal. Behav. 99, 3–13. 10.1002/jeab.1 23344985 PMC3893105

[B22] Johnson C. A. Xiao L. Palmer P. Sun P. Wang Q. Wei Y.. (2008). Affective decision-making deficits, linked to a dysfunctional ventromedial prefrontal cortex, revealed in 10th grade Chinese adolescent binge drinkers. Neuropsychologia 46, 714–726. 10.1016/j.neuropsychologia.2007.09.012 17996909 PMC3498846

[B23] Jupp B. Dalley J. W. (2014a). Behavioral endophenotypes of drug addiction: etiological insights from neuroimaging studies. Neuropharmacology 76, 487–497. 10.1016/j.neuropharm.2013.05.041 23756169

[B24] Jupp B. Dalley J. W. (2014b). Convergent pharmacological mechanisms in impulsivity and addiction: insights from rodent models. Br. J. Pharmacol. 171, 4729–4766. 10.1111/bph.12787 24866553 PMC4209940

[B25] Kelley A. E. (2004). Memory and addiction: shared neural circuitry and molecular mechanisms. Neuron 44, 161–179. 10.1016/j.neuron.2004.09.016 15450168

[B26] Leblond M. Sukharnikova T. Yu C. Rossi M. A. Yin H. H. (2014). The role of pedunculopontine nucleus in choice behavior under risk. Eur. J. Neurosci. 39, 1664–1670. 10.1111/ejn.12529 24617747 PMC4137405

[B27] Littlefield A. K. Sher K. J. (2010). The multiple, distinct ways that personality contributes to alcohol use disorders. Soc. Personal. Psychol. Compass 4, 767–782. 10.1111/j.1751-9004.2010.00296.x 21170162 PMC3002230

[B28] Lovic V. Saunders B. T. Yager L. M. Robinson T. E. (2011). Rats prone to attribute incentive salience to reward cues are also prone to impulsive action. Behav. Brain Res. 223, 255–261. 10.1016/j.bbr.2011.04.006 21507334 PMC3119757

[B29] Marinelli M. McCutcheon J. E. (2014). Heterogeneity of dopamine neuron activity across traits and states. Neuroscience 282, 176–197. 10.1016/j.neuroscience.2014.07.034 25084048 PMC4312268

[B30] McClory A. J. Spear L. P. (2014). Effects of ethanol exposure during adolescence or in adulthood on Pavlovian conditioned approach in Sprague-Dawley rats. Alcohol 48, 755–763. 10.1016/j.alcohol.2014.05.006 25449366 PMC4254554

[B31] McMurray M. S. Amodeo L. R. Roitman J. D. (2014). Effects of voluntary alcohol intake on risk preference and behavioral flexibility during rat adolescence. PLoS One 9:e100697. 10.1371/journal.pone.0100697 25007338 PMC4090063

[B32] Mejia-Toiber J. Boutros N. Markou A. Semenova S. (2014). Impulsive choice and anxiety-like behavior in adult rats exposed to chronic intermittent ethanol during adolescence and adulthood. Behav. Brain Res. 266, 19–28. 10.1016/j.bbr.2014.02.019 24566059 PMC4005391

[B33] Meyer P. J. Lovic V. Saunders B. T. Yager L. M. Flagel S. B. Morrow J. D.. (2012). Quantifying individual variation in the propensity to attribute incentive salience to reward cues. PLoS One 7:e38987. 10.1371/journal.pone.0038987 22761718 PMC3382216

[B34] Milton A. L. Everitt B. J. (2012). The persistence of maladaptive memory: addiction, drug memories and anti-relapse treatments. Neurosci. Biobehav. Rev. 36, 1119–1139. 10.1016/j.neubiorev.2012.01.002 22285426

[B35] Mitchell J. M. Fields H. L. D’Esposito M. Boettiger C. A. (2005). Impulsive responding in alcoholics. Alcohol. Clin. Exp. Res. 29, 2158–2169. 10.1097/01.alc.0000191755.63639.4a 16385186

[B36] Nasrallah N. A. Clark J. J. Collins A. L. Akers C. A. Phillips P. E. Bernstein I. L. (2011). Risk preference following adolescent alcohol use is associated with corrupted encoding of costs but not rewards by mesolimbic dopamine. Proc. Natl. Acad. Sci. U S A 108, 5466–5471. 10.1073/pnas.1017732108 21402915 PMC3069180

[B37] O’Brien C. P. Childress A. R. Ehrman R. Robbins S. J. (1998). Conditioning factors in drug abuse: can they explain compulsion? J. Psychopharmacol. 12, 15–22. 10.1177/026988119801200103 9584964

[B38] Olmstead M. C. (2006). Animal models of drug addiction: where do we go from here? Q. J. Exp. Psychol. 59, 625–653. 10.1080/17470210500356308 16707354

[B39] Olson E. A. Hooper C. J. Collins P. Luciana M. (2007). Adolescents′ performance on delay and probability discounting tasks: contributions of age, intelligence, executive functioning, and self-reported externalizing behavior. Pers. Individ. Dif. 43, 1886–1897. 10.1016/j.paid.2007.06.016 18978926 PMC2083651

[B40] Pan W. X. Hyland B. I. (2005). Pedunculopontine tegmental nucleus controls conditioned responses of midbrain dopamine neurons in behaving rats. J. Neurosci. 25, 4725–4732. 10.1523/JNEUROSCI.0277-05.2005 15888648 PMC6724780

[B41] Pascual M. Boix J. Felipo V. Guerri C. (2009). Repeated alcohol administration during adolescence causes changes in the mesolimbic dopaminergic and glutamatergic systems and promotes alcohol intake in the adult rat. J. Neurochem. 108, 920–931. 10.1111/j.1471-4159.2008.05835.x 19077056

[B42] Richards J. B. Zhang L. Mitchell S. H. de Wit H. (1999). Delay or probability discounting in a model of impulsive behavior: effect of alcohol. J. Exp. Anal. Behav. 71, 121–143. 10.1901/jeab.1999.71-121 10220927 PMC1284697

[B43] Rowland N. E. Nasrallah N. Robertson K. L. (2005). Accurate caloric compensation in rats for electively consumed ethanol-beer or ethanol-polycose mixtures. Pharmacol. Biochem. Behav. 80, 109–114. 10.1016/j.pbb.2004.10.010 15652386

[B44] Salamone J. D. Correa M. (2012). The mysterious motivational functions of mesolimbic dopamine. Neuron 76, 470–485. 10.1016/j.neuron.2012.10.021 23141060 PMC4450094

[B45] Schindler A. G. Soden M. E. Zweifel L. S. Clark J. J. (2016). Reversal of alcohol-induced dysregulation in dopamine network dynamics may rescue maladaptive decision-making. J. Neurosci. 36, 3698–3708. 10.1523/JNEUROSCI.4394-15.2016 27030756 PMC4812130

[B46] Schindler A. G. Tsutsui K. T. Clark J. J. (2014). Chronic alcohol intake during adolescence, but not adulthood, promotes persistent deficits in risk-based decision making. Alcohol. Clin. Exp. Res. 38, 1622–1629. 10.1111/acer.12404 24689661 PMC4047126

[B47] Shaham Y. Shalev U. Lu L. De Wit H. Stewart J. (2003). The reinstatement model of drug relapse: history, methodology and major findings. Psychopharmacology 168, 3–20. 10.1007/s00213-002-1224-x 12402102

[B48] Spear L. P. (2000). The adolescent brain and age-related behavioral manifestations. Neurosci. Biobehav. Rev. 24, 417–463. 10.1016/s0149-7634(00)00014-2 10817843

[B49] Spoelder M. Tsutsui K. T. Lesscher H. M. Vanderschuren L. J. Clark J. J. (2015). Adolescent alcohol exposure amplifies the incentive value of reward-predictive cues through potentiation of phasic dopamine signaling. Neuropsychopharmacology 40, 2873–2885. 10.1038/npp.2015.139 25971592 PMC4864623

[B51] Stevens L. Verdejo-Garcia A. Goudriaan A. E. Roeyers H. Dom G. Vanderplasschen W. (2014). Impulsivity as a vulnerability factor for poor addiction treatment outcomes: a review of neurocognitive findings among individuals with substance use disorders. J. Subst. Abuse Treat. 47, 58–72. 10.1016/j.jsat.2014.01.008 24629886

[B50] St Onge J. R. Floresco S. B. (2009). Dopaminergic modulation of risk-based decision making. Neuropsychopharmacology 34, 681–697. 10.1038/npp.2008.121 18668030

[B52] van Gaalen M. M. van Koten R. Schoffelmeer A. N. Vanderschuren L. J. (2006). Critical involvement of dopaminergic neurotransmission in impulsive decision making. Biol. Psychiatry 60, 66–73. 10.1016/j.biopsych.2005.06.005 16125144

[B53] Winstanley C. A. Olausson P. Taylor J. R. Jentsch J. D. (2010). Insight into the relationship between impulsivity and substance abuse from studies using animal models. Alcohol. Clin. Exp. Res. 34, 1306–1318. 10.1111/j.1530-0277.2010.01215.x 20491734 PMC3380443

