# Supplementary material for: Maladaptive Decision Making in Adults with a History of Adolescent Alcohol use, in a Preclinical Model, Is Attributable to the Compromised Assignment of Incentive Value during Stimulus-Reward Learning
Source: Front Behav Neurosci. 2017 Jul 25;11:134. doi: 10.3389/fnbeh.2017.00134 (PMC5524919; doi:10.3389/fnbeh.2017.00134)
Supplement: Supplementary file 1 [file Data_Sheet_1.docx]

Supplementary Material

**Maladaptive decision making in adults with a history of adolescent alcohol use, in a preclinical model, is attributable to the compromised assignment of incentive value during stimulus-reward learning**

Lauren C Kruse^1^, Abigail G Schindler^1,2^, Rapheal G Williams^3^, Sophia J Weber^1^, Jeremy J Clark^1^*

^1^Department of Psychiatry and Behavioral Sciences, University of Washington, Seattle, WA, USA

^2^ Geriatric Research Education and Clinical Center, VA Puget Sound Health Care System, Seattle, WA, USA

^3^Graduate Program in Neuroscience, University of Washington, Seattle, WA, USA

Corresponding Author:

Dr. Jeremy J Clark, PhD

Department of Psychiatry and Behavioral Sciences

University of Washington

1959 NE Pacific St, Box

Seattle WA 98103

Email: [jjc1@uw.edu](mailto:jjc1@uw.edu)

# Supplementary Data

**Methods**

**Effort Discounting**

Animals underwent the same 20-day continuous access to 10% alcohol gels and withdrawal period as described in the Materials and Methods section in the main text. Following instrumental training as outlined in the main text, a separate group of animals were tested on a concurrent instrumental task involving the presentation of two levers. Each daily session consisted of 24 forced trials followed by 24 free-choice trials, with a total of 5 testing sessions. At the start of each session, the chamber was in the inter-trial interval state, completely dark with no light cues. All trials began with illumination of the house light and a light in the food tray cueing the animal to make a nosepoke into the food tray within ten seconds. Failure to nosepoke resulted in trial termination, and the chamber returned to the inter-trial interval state. During training, animals were exposed to forced trials wherein a successful nosepoke led to the extension of a single lever, presented pseudo-randomly. These trials served to expose the animal to each option and its associated expected value. During each session, forced choice trials were followed by free-choice trials with the same contingency for each lever. Free-choice trials follow the guidelines described above, but each successful nosepoke resulted in the extension of both levers, and the animal was free to choose between the two levers within ten seconds. During both forced- and free-choice trials, one lever was associated with the delivery two sucrose pellets following a four lever presses and the other lever associated with the delivery of four sucrose pellets after an increasing number of lever presses (either 4, 8, 16, 32, and 64). Increasing amount of effort (lever presses) required were tested in separate testing sessions (days) with 4 lever presses on the first day of testing, 8 lever presses on the second day of testing, and so on for all animals. This method of testing the different delay times on separate days was done in order to be kept consistent with our previous studies using the probability discounting task where the different probabilistic deliveries of reward were testing on subsequent days. Each session assessed the animal’s preference between the two levers. Choice of the high reward lever was recorded during free-choice trials.

**Results**

The effort-discounting task was used to assess the effect of adolescent alcohol intake on effortful choice in adulthood. Analysis of choice behavior on the effort-discounting task generated standard discounting curves for choice of the larger but more effortful reward option over all conditions, with increasing number of lever presses required to receive the large reward resulting in decreased choice of the large reward option for both alcohol-exposed and control animals (F_(4,56) delay_ = 55.5, p < 0.0001; Supplementary Figure 1). Alcohol-exposed and control animals did not differ in choice behavior over any of the lever press conditions (F_(1, 56) treatment_ = 0.4, NS; F_(4, 66) delay x treatment_ = 0.8, NS; Supplementary Figure 1). These data indicate that moderate adolescent alcohol intake does not appear to alter effortful choice in adulthood compared to animals without a history of adolescent alcohol.

# Supplementary Figures

## Supplementary Figure


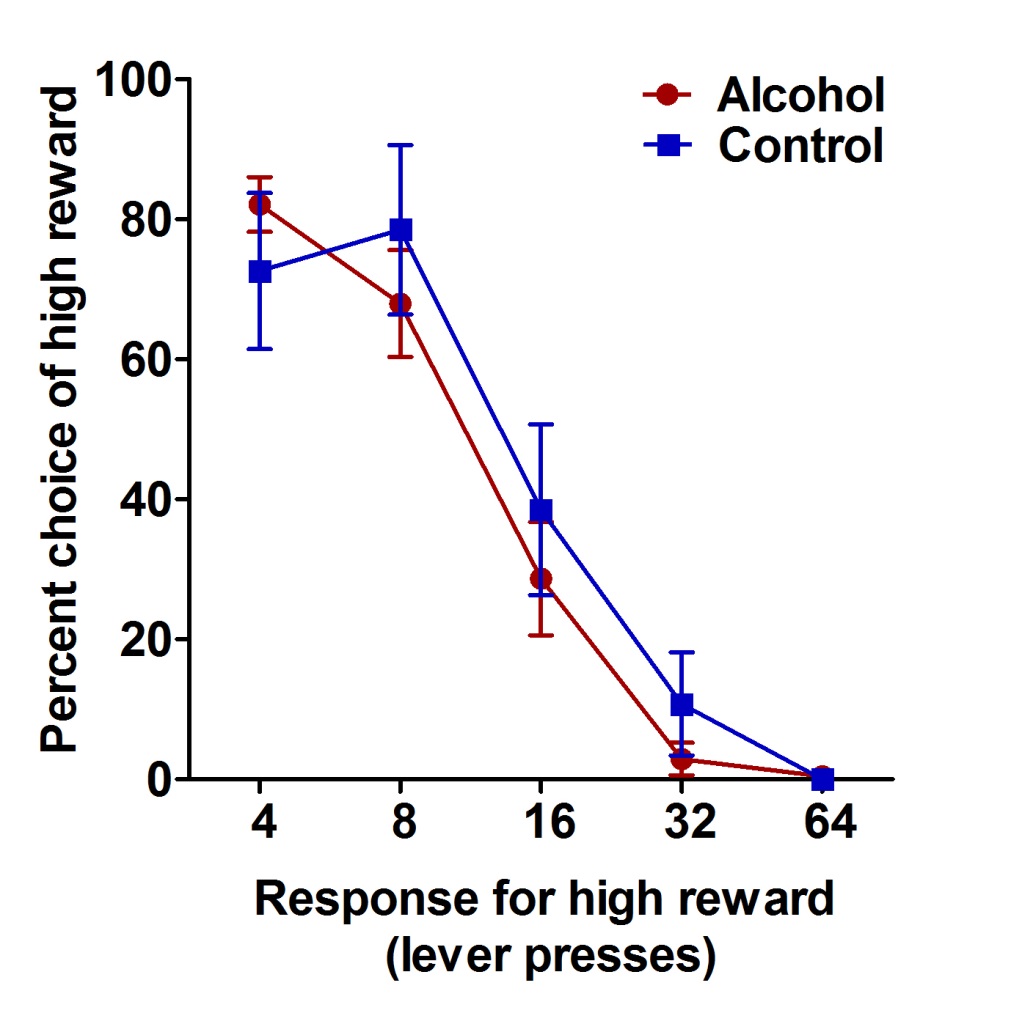


**Supplementary Figure 1.** Effortful choice as measured by performance on the effort-discounting task. Alcohol-exposed (n = 9) and control (n = 7) animals both demonstrated a decrease in choice of the large reward option with increasing amount of work required. Alcohol-exposed and control animals did not differ in choice behavior over lever press conditions. All data are presented as mean ± SEM for the percent of trials on which the larger delayed reward was chosen for 4, 8, 16, 32, and 64 lever press requirements.
